# Peri-orbital Masson’s tumor: a case report and literature review

**DOI:** 10.11604/pamj.2022.42.147.29078

**Published:** 2022-06-23

**Authors:** Lamia Benantar, Sarah Belghmaidi, El Mehdi Hamidi, Yassine Ait M'barek, Yassine El Ghani, Ibtissam Hajji, Abdeljalil Moutaouakil, Khalid Aniba

**Affiliations:** 1 Neurosurgery Department of Ibn Tofail Hospital, Mohammed VI UHC, Marrakech, Morocco,; 2 Ophtalmology Department of Arrazi Hospital, Mohammed VI UHC, Marrakech, Morocco

**Keywords:** Masson tumor, intravascular papillary endothelial hyperplasia, orbital, management, case report

## Abstract

Masson tumor is a benign vascular lesion characterized by an intravascular papillary endothelial hyperplasia. Peri orbital locations are rare. We report a case of Masson tumor localized in the upper internal angle of the left orbit revealed by progressive ocular proptosis. Orbital computed tomography (CT) scan and magnetic resonance imaging (MRI) showed a vascular mass in the left internal canthus mimicking an arterioveinous malformation. The patient underwent total removal of the lesion with a favorable postoperative follow up. Histological examination found an intravascular papillary endothelial hyperplasia without atypical features corresponding to Masson tumor. A thorough literature review of Masson tumor is presented with a discussion of clinical findings and management.

## Introduction

Masson´s tumor, also known as intravascular papillary endothelial hyperplasia (IPEH), is a rare benign neoplasm that affects the head, neck, and upper extremities [1]. Although previously described as a tumor, it is now considered as a reactive hyperplastic proliferation of vascular endothelium [2]. It typically presents as a painless, reddish purple lesion in the sites affected. The orbit remains an uncommon site of affectation of this relatively common disease, few orbital and eyelid cases have been reported. Masson´s tumor can often be difficult to distinguish between other entities. Magnetic resonance imaging (MRI) is the preferred imaging technique for identifying IPEH. Management depends on the progression of the tumor, surgical excision is the desirable method of treatment [1,2].

## Patient and observation

**Patient information:** we present the case of a 58-year-old women, with a personal medical history of intra-orbital tumor operated 10 years ago without documentation.

**Timeline:** she had been presenting a slowly progressive, painless left eye proptosis evolving for one year before admission, with no loss of visual acuity nor diplopia.

**Figure 1:**
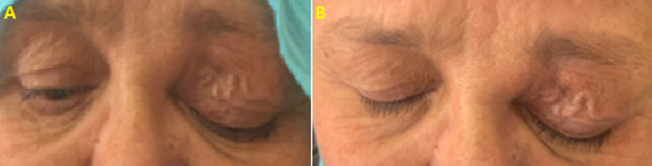
(A,B) clinical examination showing impairment and varix of upper left eyelid

**Figure 2:**
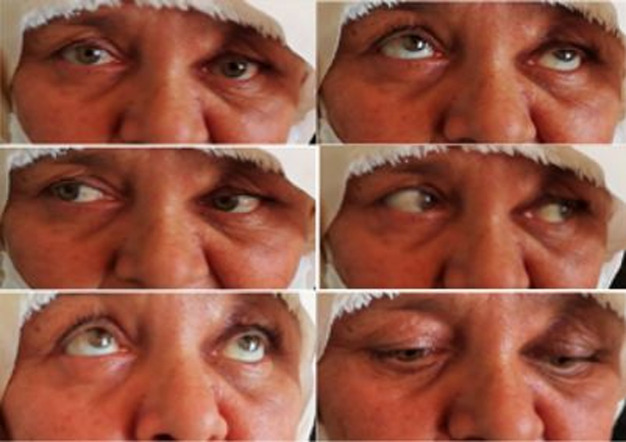
pre-operative clinical examination showing mildly restricted ocular motility

**Clinical finding:** clinical examination revealed a left non-axial proptosis with hypotropia. Palpation showed reducible, soft and swinging mass, at the upper internal angle of the orbit, with impairment and varix of upper eyelid (Figure 1), ocular motility was mildly restricted (Figure 2). The best corrected visual acuity was 0.3 logMAR in the right eye and 0.1 logMAR in the left eye, anterior segment examination revealed a cortical cataract in both eyes. Fundus examination was normal. We did not suspect any other locations of the tumor on clinical examination.

**Figure 3:**
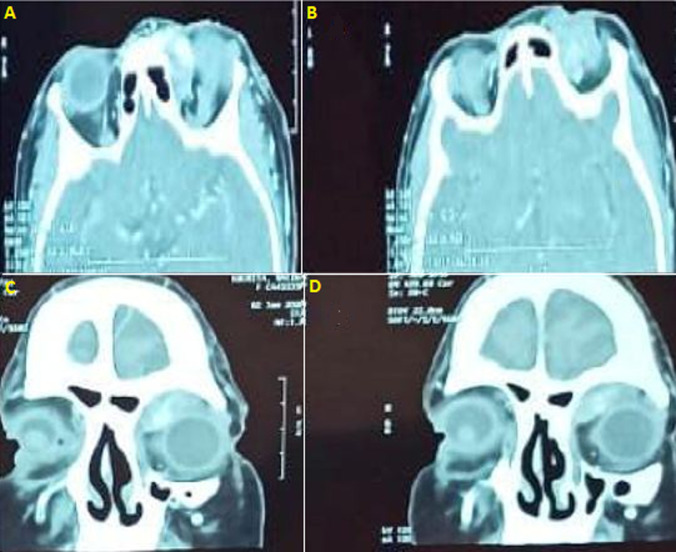
orbital CT scan with enhancement: (A,B) axial section; (C,D) coronal section, showing vascular lesion of the upper internal angle of left orbit

**Figure 4:**
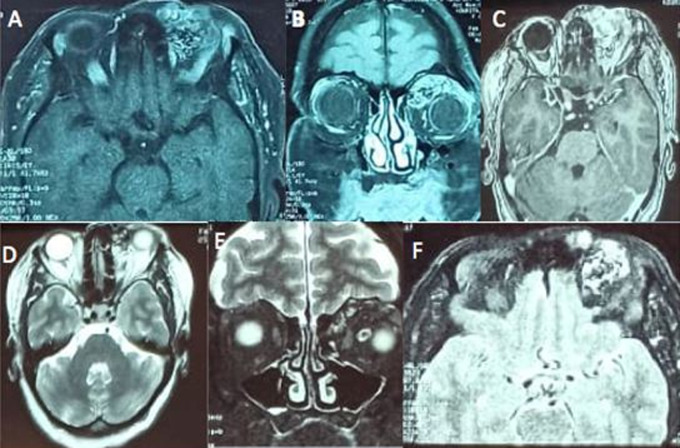
(A) orbito encephalic MRI; (B) axial and coronal T1 section: image of orbital mass in upper internal angle of left orbit, the lesion has an in heterogeneous hypersignal with serpiginous appearance; (C) axial T1 section with gadolinium enhancement, the mass has intense contrast enhancement; (D, E, F) axial and coronal T2 section, axial diffusion section: heterogeneous hypersignal lesion with serpiginous appearance mimicking an arteriovenous malformation

**Diagnostic assessment:** computed tomography scan of the orbit showed a vascular lesion of the upper internal angle of the left orbit (Figure 3). Magnetic resonance imaging (MRI) revealed an orbital vascular mass of the left internal canthus, measuring 3 x 2.7 x 2.4 cm in diameter and responsible for a grade II proptosis with scalloping on the internal orbital wall (Figure 4).

**Therapeutic intervention:** the patient underwent a total surgical removal of the tumor through a superior infra-frontal approach. The resected tumor measured 2.6 cm x 0.6 cm. the patient also received symptomatic postoperative medical treatment made of analgesics, antibiotic prophylaxis and corticosteroids for 5 days.

**Figure 5:**
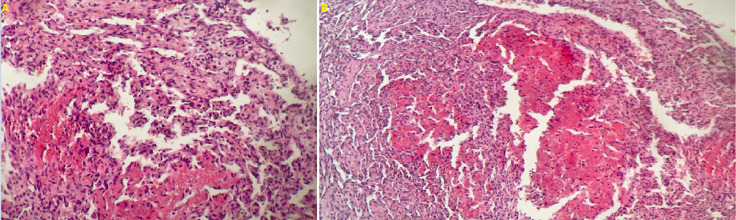
(A,B) histological examination finding endothelial cells with solid area of fibrosis and papillary hyperplasia limited to vascular wall without atypical features

**Follow up and outcomes:** post-operative follow up was simple, pathological examination revealed an intravascular papillary endothelial hyperplasia also known as Masson tumor (Figure 5).

**Patient perspective:** surgical approach and prognosis have been explained to the patient.

**Informed consent:** it was obtained from the patient so that we can use her photos as well as clinical information and radiological images for this case report.

## Discussion

Masson tumor is a common begin vascular disorder that involves dermis and subcutaneous tissue proliferation. It is known by various names: Masson´s hemangioma, Masson intravascular hemangio-endothelioma, intravascular papillary endothelial hyperplasia (IPEH), reactive papillary endothelial hyperplasia [3]. It was described by Pierre Masson in 1923 as an intravascular vegetating hemangio-endothelioma, but the current name of IPEH was proposed by Clearkin and Enzinger in 1976 [3,4]. Masson tumor was considered originally as a true neoplasm, defined recently as a reactive vascular proliferation of endothelial cells associated with an organization and a recanalization of thrombi following a traumatic vascular stasis. Still, some authors continue to consider IPEH to be a true neoplasm [1,3,5]. It can be located in extremities, breast, thyroid gland, external jugular vein, tongue, lip, oral cavity, neck, paranasal sinus and rarely intracranially (a case of facial nerve palsy). Most common locations of Masson tumor include fingers, head and neck, trunk body, lower extremities than upper extremities. Ocular evolvement is unusual (less than 20 cases reported in ophthalmologic literature) and the eyelids are typically affected in orbital lesions [1,3-5]. Intravascular papillary endothelial hyperplasia (IPEH) represents 2% of begin and malignant vascular tumor of skin and subcutaneous tissues [3]. There is no gender, racial or age preferences [3], but some authors claimed in their series a mean age of 41.2 for peri-orbital IPEH, and a sex ratio is 2.1 [1].

Clinical presentation depends on their location, and most cases are developed within vascular channels of deep dermis or subcutis [2]. The mass is painless, firm, cystic, mobile with reddish purple swelling [3,4]. In orbital locations, it can cause acute or slow onset proptosis depending on the underlying thrombosis or hematoma [1]. There are 3 types of IPEH [1,4]: pure or primary: developed within dilated vascular spaces with a preferred location in extremities skeletal muscle mass; mixed form: reactive or secondary IPEH; most common, usually intramuscular without any preferred location. Characterized by the presence of a pre-existing vascular malformation like a cavernoma, a hemangioma, a varix, a lymphongioma or a pyogenic granuloma; tertiary: exceptional, extravascular form of IPEH originating within a hematoma. Histologically, it is an intravascular proliferation of numerous papillae composed of connected tissue to endothelial surface [3,6]. The differences between these tumors and malignant lesions are that they are well circumscribed or encapsulated with a specified papillary depth in addition to the presence of a proliferating tuft structure and thrombotic material [1,4]. The proliferation process is entirely limited by a vascular wall. There are no malignant features; no atypic elements, mitotic activity, necrosis, nuclear pleomorphism, nor invasion of adjacent tissue [1,3]. Magnetic resonance imaging (MRI) is the preferred technique for identifying IPEH. The lesion is iso intense in T1 and hyper-intense in T2, with homogenous enhancement following contrast injection [5]. This enhancement on CT scan or MRI can mimick high-grade tumors [6]. Management of Masson tumor depends on clinical features and progression of the lesion (1.4). In some localisations, the tumor may spontaneously regress over time [6]. Surgical excision with complete removal is performed to prevent tumor recurrence [1,4]. Chemotherapy or radiotherapy can be proposed for recurrence after incomplete excision, multiple intracranial lesions or suspected lesions [1,4]. Differential diagnosis include vascular lesions especially malignant angiosarcoma, angiolymphatic hyperplasia with eosinophilia and other primary or secondary malignant orbital tumor [1,3,4]. Angiosarcoma is the most common differential diagnosis, it´s a lesion that invades tissues beyond one to two endothelial layers of vascular channels more than covering papillary formation with malignant features on cytology [3].

## Conclusion

Orbital localisation of Masson´s tumor or IPEH is rare. It´s a benign tumor that can mimick benign or malignant tumor or vascular malformation. Histological examination confirms the diagnosis, and the treatment of choice is complete surgical excision.

## References

[1] Wagh VB, Kyprianou I, Burns J, Brown LJR, Vaidhyanath R (2011). Periorbital Masson´stumor: a case series. Ophtalmic Plast Reconstruct Surg.

[2] Font RL, Wheeler TM, Boniuk M (1983). Intravascular papillary endothelial hyperplasia of the orbit and ocular adnexa: a report of five cases. Arch Ophthalmol.

[3] Fasina O, Adeoye A, Akang E (2012). Orbital intravascular papillary endothelial hyperplasia in a Nigerian child: a case report and review of the literature. Journal of Medical Case Reports.

[4] Dryden SC, Marsili S, Meador AG, Randall MB, Fowler B (2019). Intravascullar papillary endothelial hyperplasia of the orbit: a case of Masson´s tumor. Cureus.

[5] Aggarwal E, Madge SN, Rodgers N, Selva D (2010). Compressive effects of intravascular papillary endothelial hyperplasia. Ophtalmic Plast Reconstrr Surg.

[6] Shih SC, Burgett R, Bonnin J, Boaz J, Ho CY (2012). Intracranial Masson tumor: case report and literature review. J Neuro oncol.

